# Thermal increase in the oral mucosa and in the jawbone during Nd:YAG laser applications. Ex vivo study

**DOI:** 10.4317/medoral.17726

**Published:** 2012-02-09

**Authors:** Paolo Vescovi, Elisabetta Merigo, Carlo Fornaini, Jean P. Rocca, Samir Nammour

**Affiliations:** 1 DDS, MSc. Professor, Unit of Oral Medicine, Oral Pathology and Laser-Assisted Oral Surgery - Department of ENT-Dental Ophtalmological and Cervico-Facial Sciences - University of Parma - Italy; Doctoral school in dental sciences - Faculty of Medicine - University of Liège - Liège - Belgium; 2 DDS, MSc. Consultant Professor, Unit of Oral Medicine, Oral Pathology and Laser-Assisted Oral Surgery - Department of ENT-Dental Ophtalmological and Cervico-Facial Sciences - University of Parma - Italy; 3 MD, DDS, MSc. Consultant Professor, Unit of Oral Medicine, Oral Pathology and Laser-Assisted Oral Surgery - Department of ENT-Dental Ophtalmological and Cervico-Facial Sciences - University of Parma - Italy; Doctoral school in dental sciences - Faculty of Medicine - University of Liège - Liège - Belgium; 4 DDS, PhD, MSc . Professor, TELEO Laboratory- Faculty of Dentistry - University of Nice “Sophia Antipolis” - Nice - France; 5 DDS, PhD, MSc EMDOLA Professor, General Coordinator of EMDOLA (European Master Degree on Oral Laser Applications) - Doctoral school in dental sciences - Faculty of Medicine - University of Liège - Liège - Belgium

## Abstract

Objective: Literature reports bactericidal and biostimulant effects for Nd:YAG laser procedures on bone and oral mucosa but the possible overheating can cause damage to anatomical structures. The aim of the study is the evaluation of thermal increase in different levels of oral tissues: mucosa, periosteum and bone during defocused application of Nd:YAG laser at different parameters. Study Design: Superficial thermal evaluation was performed in pig jaws with a thermal camera device; deep thermal evaluation was realized by 4 thermocouples placed at a subperiosteal level and at 1,2 and 4 mm depth in the jaw bone. Laser applications of 1 minute were performed 5 times (with a pause of 1 minute) on a surface of 4 cm2 with a Nd:YAG laser (VSP mode, 320 micrometer fiber, defocused mode) with different parameters. Temperatures were recorded before and after laser applications and after each pause in order to evaluate also the thermal relaxation of tissues. Results: At submucosal level, mean thermal increase was between 1.1°C and 13.2°C, at 1 mm depth between 1.1°C and 8.5°C, at 2 mm depth between 1.1°C and 6.8°C, at 4 mm depth between 1.0°C and 5.3°C. Temperature decrease during the rest time period was variable between 0°C and 2.5°C. Conclusions: Temperatures reached during clinical procedures with parameters reported in the literature in biostimulation protocols (1.25-2 Watts) for the five minutes of application are not dangerous for biological structures. The decrease in temperature during the rest time period is less considerable in the bone in comparison to oral mucosa.

** Key words:**Nd:YAG laser, thermal increase, thermocouple, thermal camera, low level laser therapy.

## Introduction

Nd:YAG laser was developed in 1964 and represents a most widely used solid state crystal laser. This laser generates light in the near infrared region of the spectrum at 1064 nm and it has been used in dentistry since 1970 due to its wavelenght well absorbed by melanin and haemoglobin and then its optimal hemostatic effect on soft tissues. Due to its poor absorption in water, the laser penetrates deeply into the tissue, down to a depth of 4-5 mm. The laser beam transfers heat to the tissues producing a selective coagulation of blood vessels to a depth of about 7-10 mm (1). The laser beam effect on the target tissue depends on the parameters of the laser and the chemical and physical characteristics of the tissue. The energy transferred and absorbed results in tissue heating, which translates into ablation and/or coagulation on the target tissue.

The bactericidal and biostimulant properties of Nd:YAG laser, used in a non-focused way, have been reported in the literature: Nd:YAG laser device showed its bactericidal properties using relatively low parameters (1.5 W, 15 Hz) in non contact mode, but also provided experimental evidence for its role at low pulse energy and in promoting proliferation and differentiation of several cell types representative of the oral microenvironement (2-4).

The laser irradiation produces some changes in cellular metabolism probably having a photochemical mechanism with energy firstly absorbed by intracellular mitochondrial chromophores and thus converted to a metabolic one involving the respiratory cytochrome chain (5-6). The parameters reported in the literature for both oral mucosa and bone laser biostimulation are really non homogeneous and potentially connected to a temperature rise in tissue. In 2010 Bashardoust Tajali et al. (7) realized a meta-analysis on Low Level Laser Therapy (LLLT) on bone healing in animals: studies they accepted for the analysis reported a wide range of laser parameters, from 1.2 J/cm2 to 90 J/cm2 per session (7-9).

The antibacterial activity of Nd:YAG laser has not yet been determined but is mostly based on photothermal effects (10). Many studies referred a thermal increase in dental and periodontal tissues during endodontic, periodontal and restorative procedures. Previous investigations of the laser treated root canal showed thermal changes (e.g. melting and resolidification) thus indicating a strong temperature increase. The supraphysiological temperature rise during laser irradiation increases the risk of thermal damage to the surrounding periodontal ligament and bone. Some animal studies reported that the irradiation of teeth at 120 mJ for 30 sec resulted in ankylosis, cemental lysis and major bone remodelling in the periapical area (11). Nd:YAG laser applications in extracted teeth (12) at power settings of 60 mJ for 15 sec gave an increase on the root surface of 6.1°C that was assumed to be safe for the periodontal ligament. KTP (Potassium Titanyl Phosphate) laser application in extracted teeth is reported as compatible with a temperature increase being lower than 7 °C at root surface level only for an application time lower than 2 seconds (13).

Regarding pulp tissue, literature reports that in order to avoid pulp damage, temperature increase must be lower than 3°C (14): studies about Photo-activated Disinfection (PAD) of periodontal pockets and dental caries showed the possibility to produce a thermal increase lower than 3°C with energy densities of 5.46 J/cm2 and 4.87 J/cm2 (15-16).

Grönqvist et al. (10) in a microbiological study in vitro demonstrated that the uninterrupted Nd:YAG laser beam corresponding to 1,000 J cm² determines a temperature of approximately 70°C in agar plates. If the irradiation is performed in repeated cycles of fractionated trains of pulses consisting of 10 seconds of radiation at 25 Hz followed by 10 seconds of rest (563 J/cm²) the temperature rise is 28°C.

After every laser application the temperature decreases during resting period according to the Newton’s Law stating the formula: T(t) = Te+( T0-Te) e-kt , where T(t) is the temperature of the object at time t, Te is the constant temperature of the environment, T0 is the initial temperature of the object and kis a constant that depends on the materials properties of the object. This law states that the temperature rate of change of an object is proportional to the difference between its own temperature and the ambient temperature.

Many studies in the field of ear-nose-throat suggest to maintain standard parameters to avoid thermal damage during surgical procedure in the middle ear (17-18), suggesting a specific potential risk when the laser beam is applied to surfaces with different absorbent characteristics: bone, periosteum, oral mucosa, teeth, periodontal ligament, and dental restorations.

The aim of this study is to measure in an ex vivo model the increase in temperature in oral mucosa and in different levels of jaw bone during Nd:YAG laser application in a non-focused way with different parameters reported in the literature as useful in clinical practice.

## Material and Methods

A freshly extracted mandible of pig was used to carry out this study: specimens were kept at room temperature (superficial temperature of the mandible 19.1°C) and irradiated within 6 hours from the animal sacrifice: as reported in literature (19), specimens were stored during transit at 2-4°C and 100% of humidity to prevent tissue degradation. All measurements were made at room and bone temperature of 19.1°C.

**Figure 1:**
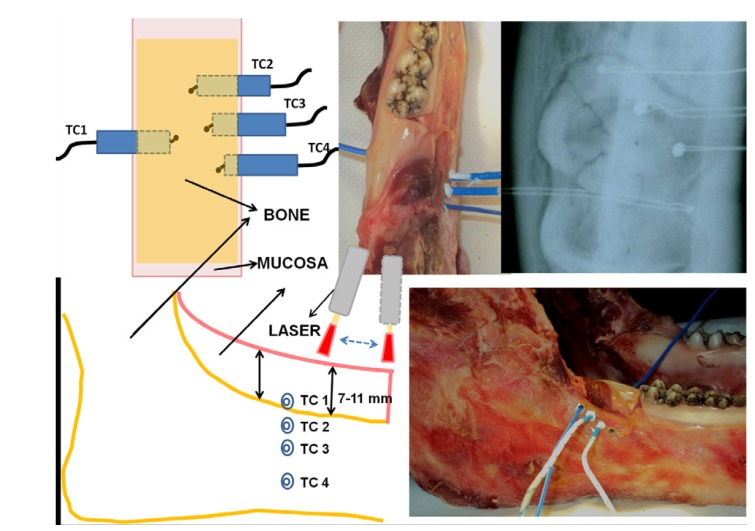
Graphical representation and pictures of placement of 4 thermocouples and X-ray control of their right position. Nd:YAG laser application and Thermal Camera monitoring of surface temperature.

In the retromolar trigonus region an area measuring 4 cm² (2 x 2 cm) was identified and circumscribed. The thickness of surrounding mucosa was between 7 and 11 mm and the thickness of bone was 2.3 cm. A mucoperiostal envelope flap was detached with a 15 c Bard Parker scalpel and the first thermocouple was inserted under the mucosa in contact with the surface of bone (TC1). With a micromotor drill three successive holes were performed in the jawbone under the previous area at 1, 2, and 4 mm and the next three thermocouples (TC2, TC3, TC4) were inserted in depth. The correct position to uniformly record temperature of each thermocouple in the middle of the bone (1.2 cm), was controlled with a X-ray (Fig. 1). The 4 naked-bead chrome-alumel (K-type) thermocouples (TP-01 - Lutron - Taiwan) had a 0.5 mm diameter probe sensitive to temperature variations between -40°C and 250°C; thermocouples were connected to a four-channel thermometer (TM-946 – Lutron – Taiwan), sensitive to temperature variations between -100°C and 1300°C, with an accuracy of 0.1°C. Surface temperature was checked during all procedures with a Thermal Camera (Thermovision A 800 - Flyr System - Sweden) connected to a PC and working with the Software ThermaCAM Researcher: thermal camera real time video allows us to check the omogeneity during the irradiation procedure, performed in a scanning mode (covering the irradiation area along vertical and horizontal lines) showing the effects on surface temperature during the action of laser device.

A thermo-conductor paste (Warme Leftpast WPN 10, Austerlitz Electronic- Germany) was spread on the walls of the created holes to ensure good thermal contact: the thermal conductivity of the paste was 0,4 cal s-1 m-1 K-1, in a way comparable with thermal conductivity of human tissues.

The laser applications were performed with a Nd:YAG Laser (1064 nm) (Fidelis Plus®; Fotona - Slovenia) in VSP (100 microsec) using a fibre of 320 μm diameter in a non-focused way at a distance of 2mm from tissue, for 1 minute in a scanning method (1mm/sec) (Fig. 1) covering in the irradiation time (1 minute) a 2 cm2 area. The irradiation of the tissue was repeated 4 successive times with 1 minute of rest after each application.

The laser parameters have been chosen on the basis of two different applications, and specifically parameters for test 1 and 2 on the basis of the literature about the Nd:YAG low level laser application on bone diseases as osteonecrosis of the jaws (20) and on vascular diseases (1), parameters for test 3 and 4 as “high level” parameters reported on different types of surgical applications (21), in order to find the “thermal increase cut off”. Parameters used in the different tests were the following:

- TEST 1: 1 W, 15Hz (PD: 1250 W/cm2 – Fluence 1125 * 105 J/cm2)

- TEST 2: 2 W, 15 Hz (PD: 2500 W/cm2 – Fluence 2250 * 105 J/cm2)

- TEST 3: 4 W, 30 Hz (PD: 5000 W/cm2 – Fluence 9000 * 105 J/cm2)

- TEST 4: 7 W, 40 Hz (PD: 8750 W/cm2 – Fluence 21000 * 105 J/cm2)

(Laser parameters were calculated on the basis of fibre diameter due to the fact that treatments were made in scanning mode: by using as surface value the 2 cm2 area of the scanning mode, power density values are 0,5 W/cm2 for test 1, 1 W/cm2 for test 2, 2 W/cm2 for test 3 and 3,5 W/cm2 for test 4).

The temperature was calculated before laser application and at the end of every irradiation including at the end of each rest time. Each test was repeated four times and for the final result we considered the mean temperature.

## Results

**Figure 2:**
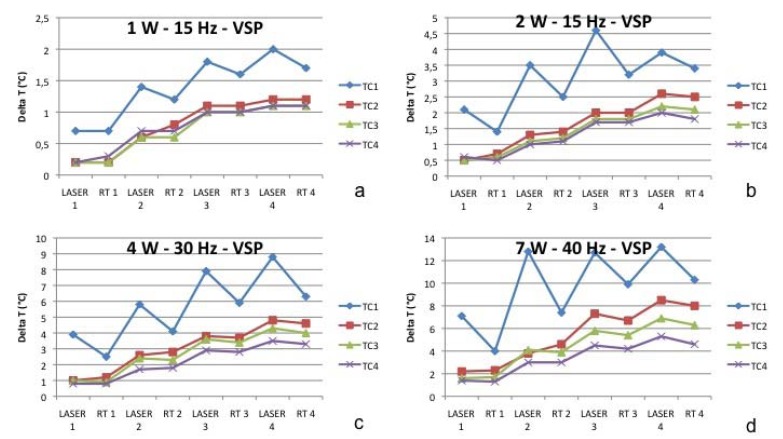
Test 1 (a), Test 2 (b), Test 3 (c), Test 4 (d). Laser: laser treatment with a duration of 1 minute; RT: Rest Time with a duration of 1 minute. TC1 = thermocouple at subperiosteal level. TC2 = thermocouple in the jawbone at 1 mm of distance from the surface of mandible. TC3= thermocouple in the jawbone at 2 mm of distance from the surface of mandible. TC4 = thermocouple in the jawbone at 4 mm of distance from the surface of mandible

**Figure 3:**
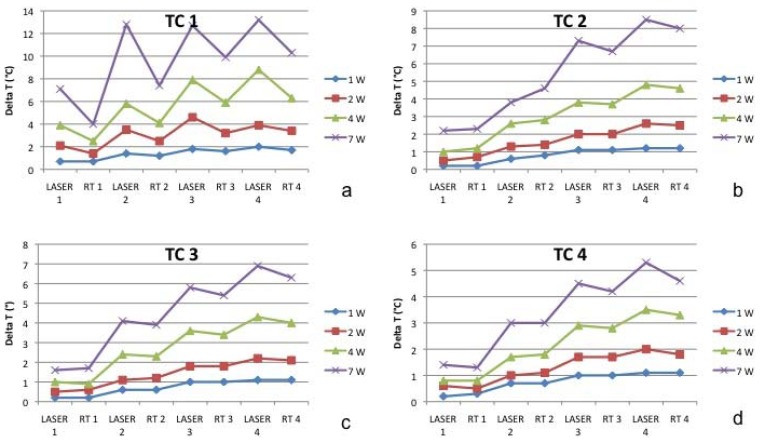
Thermal variations on the thermocouple at subperiosteal level. (TC1-a) and into the jawbone at 1 mm (TC2-b), 2 mm (TC3-c) and 4 mm (TC4-d) of distance from the surface of mandible (TC2) during Test 1, 2, 3 and 4. TC1 = thermocouple at subperiosteal level. TC2 = thermocouple in the jawbone at 1 mm of distance from the surface of mandible. TC3= thermocouple in the jawbone at 2 mm of distance from the surface of mandible. TC4 = thermocouple in the jawbone at 4 mm of distance from the surface of mandible

TEST 1: (see  Table 1 and Fig. 2-3)

**Table 1:**
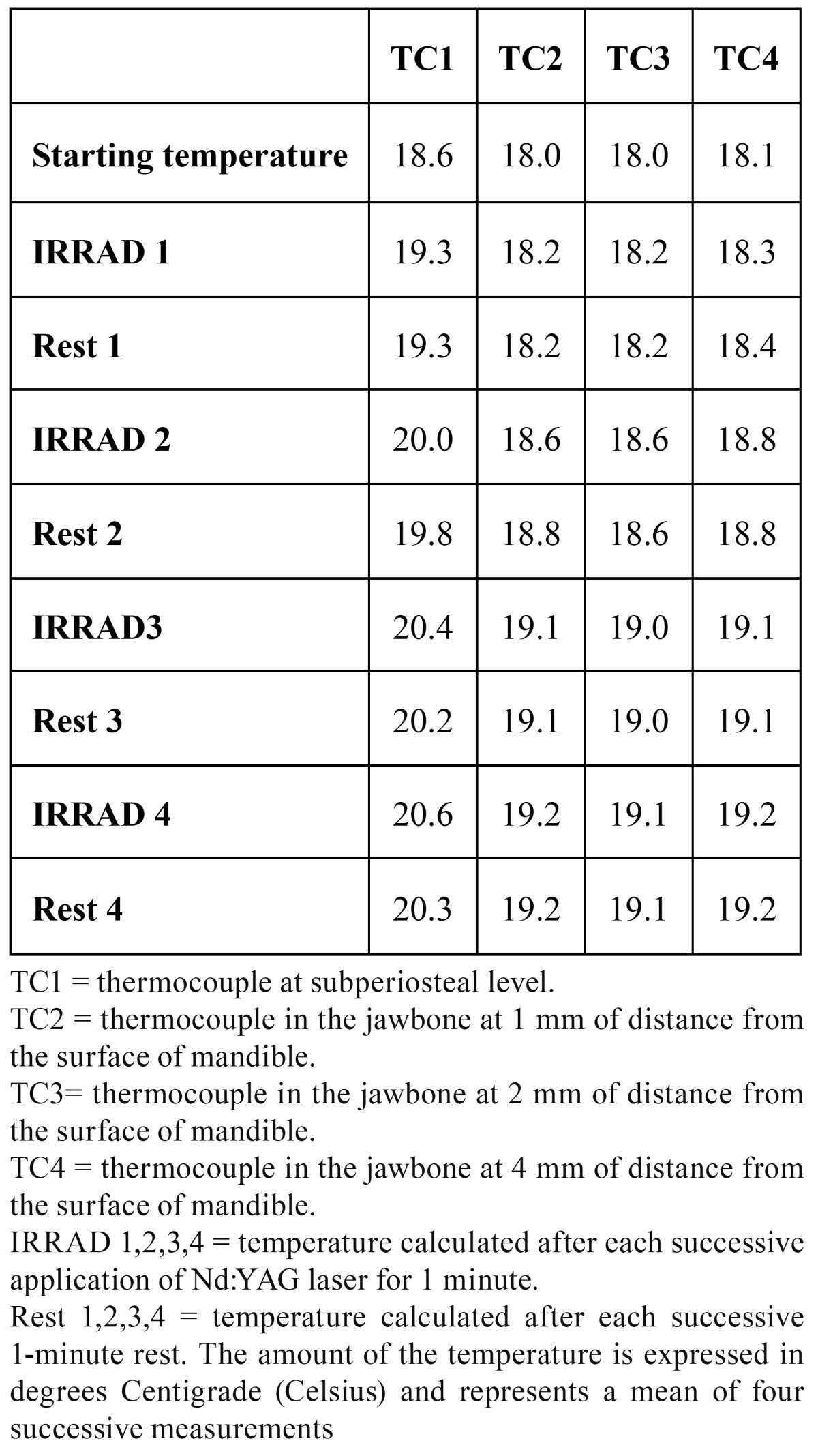
TEST 1: 1W, 15Hz.

The starting temperature was 18.6 °C in TC1, 18.0 °C in TC2, 18.0 °C in TC3, and 18.1 °C in TC4). Considering the starting temperature in TC1 after the first application the heat increased by 0.7 °C, after the second irradiation by 1.4 °C, after the third by 1.8°C and after the fourth it increased by 2.0 °C. During each rest time the temperature decreased by 0,2 °C.

In TC2 the temperature increased by 0.2 °C, 0.6 °C, 1.1 °C and 1.2 °C. During rest time, it was quite stable. In TC3 the temperature increased by 0.2 °C, 0.6 °C, 1.0 °C and 1.1 °C. During rest time, it was absolutely stable (no decrease in comparison to the amount of heat reached after each irradiation). In TC3 the temperature increased by 0.2 °C, 0.5 °C, 1.0 °C and 1.1 °C. During rest time, it was absolutely stable.

TEST 2: (see  Table 2 and Fig. 2-3)

**Table 2:**
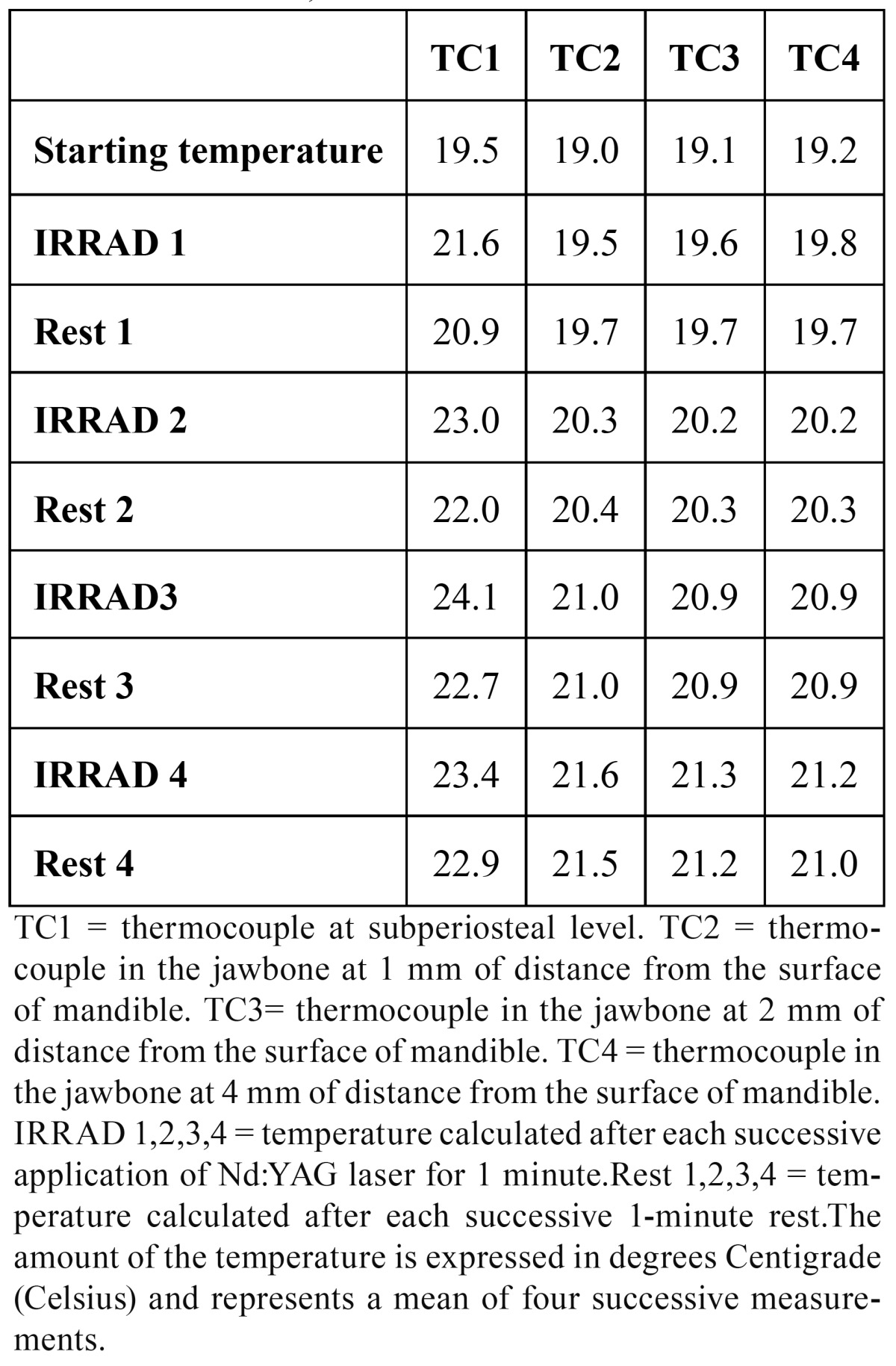
TEST 2: 2W, 15Hz.

The starting temperature in TC1 was 19.5 °C, in TC2 19.0 °C, in TC3 19.1 °C and in TC4 19.2 °C. In TC1 after the first application the heat increased by 1.1 °C, after the second irradiation by 3.5 °C, after the third by 4.6°C and after the fourth it increased by 3.9 °C. During each rest time the temperature decrease ranged between 0.7 °C and 1.4 °C. In TC2 the temperature increased by 0.5 °C, 1.3 °C, 2.0 °C and 1.6 °C. During rest time it was quite stable. In TC3 the temperature increased by 0.5 °C, 0.6 °C, 1.2 °C and 2.2 °C. During rest time it was absolutely stable (no decrease in comparison to the amount of heat reached after each irradiation). In TC3 the temperature increased by 0.6 °C, 1.0 °C, 1.7 °C and 2.0 °C. During rest time it was absolutely stable.

TEST 3: (see  Table 3 and Fig. 2-3)

**Table 3:**
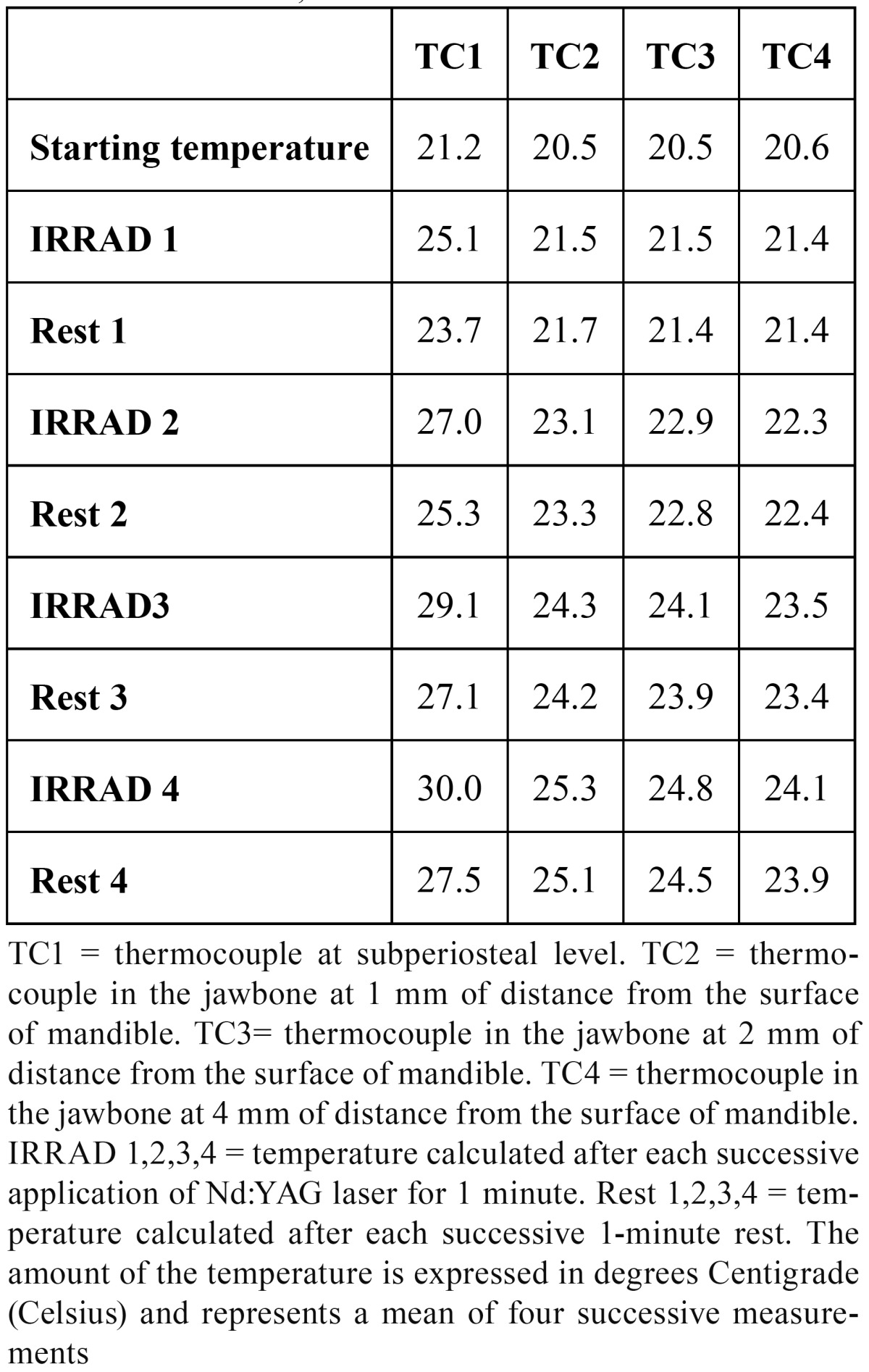
TEST 3: 4W, 30Hz.

The starting temperature in TC1 was 21.2 °C, in TC2 20.5 °C, in TC3 20.5 °C and in TC4 20.6 °C. In TC1 after the first application the heat rose by 3.9 °C, after the second irradiation by 5.8 °C, after the third by 7.9 °C and after the fourth by 8.8 °C. During each rest time the temperature decreased by about 2.5 °C after 1 minute of each irradiation. In TC2 the temperature increased by 1.0 °C, 2.6 °C, 3.8 °C and 4.8 °C. During rest time it was quite stable: the temperature decreased by 0.2 °C.

In TC3 the temperature increased by 1.0 °C, 2.4 °C, 3.6 °C and 4.3 °C. During rest time it was stable (a mean decrease of 0.1 °C). In TC3 the temperature increased by 0.8 °C, 1.7 °C, 2.9 °C and 3.5 °C. During rest time it was stable (a mean decrease of 0.1 °C).

TEST 4: (see  Table 4 and Fig. 2-3)

**Table 4:**
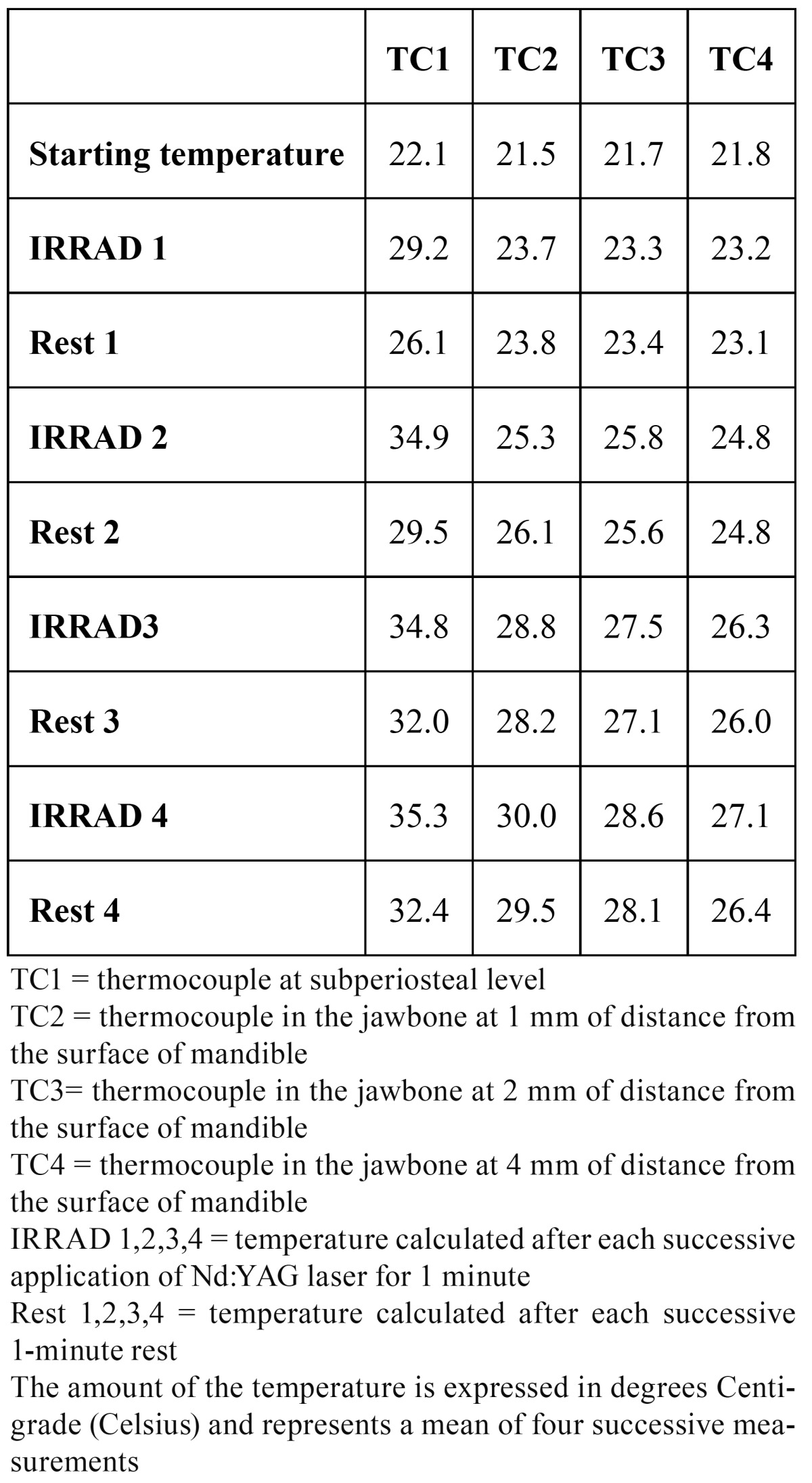
TEST 4: 7W, 40Hz.

The starting temperature in TC1 was 22.1 °C, in TC2 21.5 °C, in TC3 21.7 °C and in TC4 21.8 °C. In TC1 after the first application the heat rose by 7.1 °C, after the second irradiation by 12.8 °C, after the third by 12.7 °C and after the fourth by 13.2 °C. During the subsequent rest time the temperature decreased by 3.1 °C, 5.4 °C, 2.8 °C and 2.9 °C respectively.

In TC2 the temperature increased by 2.2 °C, 3.8 °C, 7.3 °C and by 8.5 °C. During rest time it was quite stable: the temperature decreased by a mean of 0.2 °C.

In TC3 the temperature increased by 1.6 °C, 4.1 °C, 5.8 °C and by 6.9 °C. During rest time it was stable (a mean decrease of 0.1 °C).

In TC3 the temperature increased by 1.4 °C, 3.0 °C, 4.5 °C and by 5.3 °C. During rest time it was stable (a mean decrease of 0.2 °C).

Statistical analysis by Kruskal-Wallis test (non-parametric ANOVA) showed a statistically significant difference among the 4 thermocouples in test 2, 3 and 4 with very significant differences between TC 1 and TC 4 for thermal variations at lasering time (p=0.0178, p=0.0063, and p=0.0117 respectively) and rest time (p=0.0237, p=0.0173, and p=0.0233 respectively).

## Discussion

The temperature of the mandible is related to the heating of the room and to the local conditions.

The starting bone temperature is not a significant factor and increase in bone temperature from room (19.1°C) to body temperature (37°C) does not change the properties of bone and does not influence the maximum temperature rise during laser treatment (22). Small differences and a slight temperature increase are related to the heat produced by the lamp, by the sample manipulation during the experiment, and by the progressive warmth accumulation after laser irradiations. A thermal camera can only record the global superficial heat of the mandible but the four-thermocouple system can check the internal variation of the warmth (23-24).

- A thermal increase was recorded even at low energies and not only on the surface but also in the bone and it was directly proportional to laser power augmentation (from test 1 to test 4).

- The temperature increase at high power is higher and much faster in surrounding mucosa than in the bone but the warmth is rapidly dissipated during rest time ( Table 4- Fig. 2).

- The temperature increase in the bone is slow but progressive and the heat is accumulated for more time and little by little dissipated.

- A 5.5 °C critical temperature increase for dental pulp vitality during laser applications in conservative, prosthetic or aesthetic dentistry (25-26) is reported in the literature. During conditioning of the dentin surface with a Nd:YAG laser (83-100 mJ/pulse, 10-20 pps and 20-180 s processing time) Mehl et al. (27) referred an intra-pulpal temperature increase of above 8 °C: for that reason he came to the conclusion that it is compulsory to avoid the use of Nd:YAG laser at high power. Cellular death caused by heat is immediately evident with temperature above 70°C (28): literature reports that temperatures of 47°C for 5 minutes, 50°C for 1 minute, or 56°C for less than 1 minute result in immediate bone necrosis (29-30).

In our experiment in the test 1 and 2 the maximum temperature rise in the mucosa was 2.0 °C and 4.6 °C and in the bone 1.1 °C and 2.2 °C; consequently it was not dangerous for healthy tissues. On the other hand in the test 3 after the fourth irradiation the temperature rose by 8.8 °C in the subperiosteal area (TC1) with a risk of heat diffusion in the underlying bone. In the test 4 we found a very high thermal increase: after the second laser irradiation temperature rose by 12.8 °C, after the third by 12.7 °C and after the fourth by 13.2 °C. During the rest time the warmth was dissipated progressively: 3.1 °C, 5.4 °C, 2.8 °C and 2.9 °C, but the temperature of mandible remained however in the critical range. While in the bone the temperature increase is less high, it is relatively near to the critical limits. After the fourth laser application the thermal rise was 8.5 °C in TC2, 6.9 °C in TC3, 5.3 °C in TC4. The warmth dissipation during the rest time was not sufficient to bring back the tissue to a safe thermal level. The heat of jawbone remained quite stable: the temperature decreased by a mean of 0.2 °C.

At high power the rest time is not sufficient to induce good warmth dissipation hence it is better to perform laser applications under a cooling system (air or water).

The temperature increase in vivo can be modified by many circumstances: blood circulation and water tissue, environment, local conditions and the interindividual differences in surrounding tissue thickness, so that the situation proves to be relatively dissimilar from an experiment ex vivo. On the other hand for the moment it is still uncertain what is the real amount of temperature increase in the oral mucosa and jawbone in clinical circumstances. In our study the samples were not rinsed during Nd:YAG laser applications and the temperature was recorded each time before, during and 1 min after each irradiation: the reasons we explained do not allow us to perfectly reproduce the in vivo conditions particularly the cooling effect of the blood flow and the hydration of tissues. The results of our test suggest that even at low energy laser beam induces a thermal modification not only in the mucosa but also in depth. At low power the thermal rise is very limited and not dangerous for healthy tissues but with growing power the temperature gradually increases and accumulates both in mucosa and bone. Defocused laser applications for biostimulation and bactericidal activities can be considered biologically compatible without risk of necrosis of jawbone and mucosa. On the other hand it is always compulsory to avoid prolonged laser application and to ensure a reasonable rest time (at least 1 minute) to dissipate warmth. Our study confirms that when using high energies a cooling system shall also be used to reduce thermal increase. The balance between the risk of irreversible side-effects and the efficacy of laser applications is closely related to a careful selection of laser parameters and a precise evaluation of the anatomical structures. Finally, this study could help to demonstrate that it is possible to obtain possible effects on biostimulant and bactericidal laser applications even maintaining temperatures compatible with safe protocols.
